# Supplementary material for: Biological Activities of Mikania glomerata and Mikania laevigata: A Scoping Review and Evidence Gap Mapping
Source: Pharmaceuticals (Basel). 2025 Apr 9;18(4):552. doi: 10.3390/ph18040552 (PMC12030693; doi:10.3390/ph18040552)
Supplement: Supplementary file 1 [file pharmaceuticals-18-00552-s001.zip › pharmaceuticals-3551913-supplementary.pdf]

Supplementary Material

Biological activity of *Mikania glomerata* and *Mikania laevigata*: a scoping review with a gap map

Thais Peregrin Garcia, Daniela Gorski, Alexandre de Fátima Cobre, Raul Edson Lazo Luna[a], Gustavo Bertol, Luana Mota Ferreira, Roberto Pontarolo

Table S1. Search strategy

Pubmed	
#**1** "Mikania"[MeSH Terms] OR ("Mikania"[All Fields] e ("laevigata"[All Fields] OR "glomerata"[All Fields])) OR "m laevigata"[All Fields] OR "m glomerata"[All Fields] OR "guaco"[All Fields] OR (("Cumarinas"[MeSH Terms] OR "Cumarina*"[All Fields] OR "1,2-Benzopyrones"[All Fields] OR "1,2 Benzopyrones"[All Fields] OR "1,2-Benzo-Pyrones"[All Fields] OR "1,2 Benzo Pyrones"[All Fields] OR "Benzopyran"[All Fields]<br>#**2** "Plant Leaves"[MeSH Terms] OR "leaves*"[All Fields] OR "aerial part*"[All Fields])<br>#**3** "Mikania"[All Fields] OR "guaco"[All Fields]<br>#**4** "activit*"[All Fields] OR "effect*"[All Fields] OR "efficacy*"[All Fields] OR "safety"[All Fields]<br>**#1 AND #2 AND #3 AND #4**	**114**
**Scopus**	
#**1** ALL (("Mikania" AND ("laevigata" OR "glomerata")) OR "m laevigata" OR "m glomerata" OR "guaco" OR "Cumarina*" OR "1,2-Benzopyrones" OR "1,2 Benzopyrones" OR "1,2-Benzo-Pyrones" OR "1,2 Benzo Pyrones" OR "Benzopyran")<br>#**2** ALL "leaves*" OR "aerial part*"<br>#**3** ALL "Mikania" OR "guaco"<br>#**4** ALL ("activit*" OR "effect*" OR "efficacy*" OR "safety")<br>**#1 AND #2 AND #3 AND #4**	**1610**
**Web Of Science**	
#**1** ALL (("Mikania" AND ("laevigata" OR "glomerata")) OR "m laevigata" OR "m glomerata" OR "guaco" OR ((“Cumarina*” OR “1,2-Benzopyrones” OR “1,2 Benzopyrones” OR “1,2-Benzo-Pyrones” OR “1,2 Benzo Pyrones” OR “Benzopyran”)<br>#**2** ALL (“leaves*” OR “aerial part*”)<br>#**3** ALL ("Mikania" OR "guano")<br>#**4** ALL ("activit*" OR "effect*" OR “efficacy*” OR “safety”)<br><br>**#1 AND #2 AND #3 AND #4**	**140**

Table S2. Excluded studies, after full-text reading, and justifications

Studies	Justifications
Ares, G., Barreiro, C., & Gámbaro, A. (2010). Evaluation of antioxidant extracts from Uruguayan native plants: Importance of sensory characteristics. CYTA - Journal of Food, 8(3), 201–207. https://doi.org/10.1080/19476330903353536	Plant used
Fulanetti, F. B., Camargo, G. G., Ferro, M. C., R, & azzo-Moura, P. (2016). Toxic effects of the administration of Mikania glomerata Sprengel during the gestational period of hypertensive rats. *Open Vet J*, *6*(1), 23–29. https://doi.org/10.4314/ovj.v6i1.4	Part of the plant used
Della Pasqua, C. D. P., Antunes, E., Sawaya, A., Campos, R., L, & ucci, E. C. T. (2019). Beneficial effects of aqueous extracts of Mikania glomerata Sprengel and Mikania laevigata Schultz Bip ex Baker on the inflammatory response in rats with acute pancreatitis. *Faseb Journal*, *33*.	Study design
Yatsuda, R., Rosalen, P. L., Cury, J. A., Rehder, V. G., Koo, H., Cecanho, R., & Goncalves, R. B. (2001). Effect of Mikania laevigata and Mikania glomerata on mutans streptococci. Journal of Dental Research, 80(4), 1054.	Study design
Baltodano, B. B., Mora, F. C., Álvarez, L. J. P., Castro, S. J., & Rodríguez, G. (2006). Effect of etnomedically used plants on the hemorrhagic and proteolytic activity induced by Bothrops asper. Revista Cubana de Plantas Medicinales, 11(1). https://www.scopus.com/inward/record.uri?eid=2-s2.0-33845455185&partnerID=40&md5=bf3aa7d4352c6a845fa0c698e5319c11	Plant used
de Mello, F. B., & de Mello, J. R. B. (2006). Evaluation of antitussive/expectorant effects of two phyoterapic formulations existent in the brasilian market. Acta Farmaceutica Bonaerense, 25(1), 64–70. https://www.scopus.com/inward/record.uri?eid=2-s2.0-33744533623&partnerID=40&md5=61ed05b378634b33ab36565292d2c700	Plant mixture
Silveira, D., Prieto-Garcia, J. M., Boylan, F., Estrada, O., Fonseca-Bazzo, Y. M., Jamal, C. M., Magalhães, P. O., Pereira, E. O., Tomczyk, M., & Heinrich, M. (2020). COVID-19: Is There Evidence for the Use of Herbal Medicines as Adjuvant Symptomatic Therapy? *Frontiers in Pharmacology*, *11*. https://doi.org/10.3389/FPHAR.2020.581840	Study design
Saleem, S., Khan, R., Kazmi, I., & Afzal, M. (2019). Medicinal plants in the treatment of arthritis. *Plant and Human Health: Pharmacology and Therapeutic Uses*, *3*, 101–137. https://doi.org/10.1007/978-3-030-04408-4_6	Study design
Sib, a, T., & Okoh, A. I. (2007). The challenges of overcoming antibiotic resistance: Plant extracts as potential sources of antimicrobial and resistance modifying agents. African Journal of Biotechnology, 6(25), 2886–2896. https://www.scopus.com/inward/record.uri?eid=2-s2.0-38049132540&partnerID=40&md5=343737e44199b131a6ab89407de5b6d0	Study design
Botsaris, A. S. (2007). Plants used traditionally to treat malaria in Brazil: the archives of Flora Medicinal. J Ethnobiol Ethnomed, 3, 18. https://doi.org/10.1186/1746-4269-3-18	Study design
Corrêa, M. F. P., de Melo, G. O., & Costa, S. S. (2008). Natural products from plant origin potentially usefull in the asthma therapy. Revista Brasileira de Farmacognosia, 18, 785–797. https://doi.org/10.1590/S0102-695X2008000500025	Study design

Doughari, J. H., Human, I. S., Bennade, S., & Ndakidemi, P. A. (2009). Phytochemicals as chemotherapeutic agents and antioxidants: Possible solution to the control of antibiotic resistant verocytotoxin producing bacteria. Journal of Medicinal Plants Research, 3(11), 839–848. https://www.scopus.com/inward/record.uri?eid=2-s2.0-73649093118&partnerID=40&md5=920d93ad054ee28a2a8e43ce423f50e5	Study design
Napimoga, M. H., & Yatsuda, R. (2010). Scientific evidence for Mikania laevigata and Mikania glomerata as a pharmacological tool. J Pharm Pharmacol, 62(7), 809–820. https://doi.org/10.1211/jpp.62.07.0001	Study design
Guimarães, C. L. S., Moreira-Dill, L. S., Fern, es, R. S., Costa, T. R., Hage-Melim, L. I. S., Marcussi, S., Carvalho, B. M. A., da Silva, S. L., Zuliani, J. P., Fern, es, C. F. C., Calderon, L. A., Soares, A. M., & Stábeli, R. G. (2014). Biodiversity as a source of bioactive compounds against snakebites. Current Medicinal Chemistry, 21(25), 2952–2979. https://doi.org/10.2174/09298673113206660295	Study design
França, E. L., Justo, C. F., Gonzatti, M. B., & Honorio-França, A. C. (2017). Association of medicinal plants and melatonin in human health. *Serotonin and Melatonin: Their Functional Role in Plants, Food, Phytomedicine, and Human Health*, 219–246. https://doi.org/10.4324/9781498739054	Study design
Hussein, A. A., Romero, L., López-Pérez, J. L., & Gupta, M. P. (2017). 2α-Acetoxy-15-acetylartemisiifolin, a new Anti-trypanosomal Sesquiterpene Lactone from *Mikania guaco*. *Natural Product Communications*, *12*(5), 1934578X1701200. https://doi.org/10.1177/1934578X1701200504	Study design
Ushimaru, P. I., Barbosa, L. N., Fern, es, A. A., di Stasi, L. C., Fern, & es A., Jr. (2012). In vitro antibacterial activity of medicinal plant extracts against Escherichia coli strains from human clinical specimens and interactions with antimicrobial drugs. Nat Prod Res, 26(16), 1553–1557. https://doi.org/10.1080/14786419.2011.568943	Study design
Alves, Â. V. F., Melo, C. R., Chagas-Neto, J. L., Amaral, R. G., Ambrósio, S. R., Moreira, M. R., Veneziani, R. C. S., Cardoso, J. C., Severino, P., Gondak, R. O., Souto, E. B., & de Albuquerque-Júnior, R. L. C. (2023). Ent-kaurenoic acid-enriched Mikania glomerata leaves-complexed β-cyclodextrin: Pharmaceutical development and in vivo antitumor activity in a sarcoma 180 mouse model. *International Journal of Pharmaceutics*, *631*. https://doi.org/10.1016/J.IJPHARM.2022.122497	enriched extract
Zayapor, M. N., Abdullah, A., & Wan Mustapha, W. A. (2021). The antioxidant analysis and α-glucosidase inhibition activities of spices and herbs (22 species) in Asian traditional beverages. *Journal of Food Measurement and Characterization*, *15*(2), 1703–1718. https://doi.org/10.1007/s11694-020-00766-w	Plant used
Possebon, L., de Souza Lima Lebron, I., Furlan da Silva, L., Tagliaferri Paletta, J., Glad, B. G., Sant'Ana, M., Iyomasa-Pilon, M. M., Ribeiro Souza, H., de Souza Costa, S., Pereira da Silva Rodriguesa, G., Pereira, M. de L., de Haro Moreno, A., & Girol, A. P. (2018). Anti-inflammatory actions of herbal medicines in a model of chronic obstructive pulmonary disease induced by cigarette smoke. *Biomedicine & Pharmacotherapy = Biomedecine & Pharmacotherapie*, *99*, 591–597. https://doi.org/10.1016/J.BIOPHA.2018.01.106	Plant mixture
Henriques, B. O., Corrêa, O., Azevedo, E. P. C., Pádua, R. M., Oliveira, V. L. S. D., Oliveira, T. H. C., Boff, D., Dias, A. C. F., Souza, D. G. D., Amaral, F. A., Teixeira, M. M., Castilho, R. O., & Braga, F. C. (2016). In Vitro TNF- α Inhibitory Activity of Brazilian Plants and Anti-Inflammatory Effect of Stryphnodendron adstringens in an Acute Arthritis Model. *Evidence-Based Complementary and Alternative Medicine*, *2016*. https://doi.org/10.1155/2016/9872598	Part of the plant used

Gasparetto, J. C., Peccinini, R. G., de Francisco, T. M., Cerqueira, L. B., Campos, F. R., & Pontarolo, R. (2015). A kinetic study of the main guaco metabolites using syrup formulation and the identification of an alternative route of coumarin metabolism in humans. *PLoS One*, *10*(3), e0118922. https://doi.org/10.1371/journal.pone.0118922	Study design
Motta, Y. P., Sakate, M., Nogueira, R. M. B., Peraçoli, M. T. S., Sangiorgio, F., Floriano, R. S., & Takahagi, E. N. (2014). Quantification of cytokines in serum and paw homogenate of experimental intoxication for venom of the Bothropoides jararaca in Wistar rats treated with antivenom and Mikania glomerata. Arquivo Brasileiro de Medicina Veterinaria e Zootecnia, 66(5), 1413–1418. https://doi.org/10.1590/1678-6829	enriched extract
Motta YP, Sakate M, Nogueira RMB, et al. Quantification of cytokines in serum and paw homogenate of experimental intoxication for venom of the Bothropoides jararaca in Wistar rats treated with antivenom and Mikania glomerata. *Arquivo Brasileiro de Medicina Veterinaria e Zootecnia.* 2014;66(5):1413-1418. doi:10.1590/1678-6829	Not an extract
Fournet, A., Barrios, A. A., & Muñoz, V. (1994). Leishmanicidal and trypanocidal activities of Bolivian medicinal plants. Journal of Ethnopharmacology, 41(1), 19–37. https://doi.org/10.1016/0378-8741(94)90054-X	Plant used
Dorigoni, P. A., Ghedini, P. C., Baptista, K. C., Ethur, A. B. M., Baldisserotto, B., Bürger, M. E., Almeida, C. E., Lopes, A. M. v, & Záchia, R. A. (2001). Data of medicinal plants commonly used in the city of São João do Polêsine, RS, Brazil. Relationship between illness and use of species. Revista Brasileira de Plantas Medicinais, 4(1), 69–79. https://www.scopus.com/inward/record.uri?eid=2-s2.0-17144447016&partnerID=40&md5=160ed23cde67662b08d6174e1254b39a	Study design
Abe, F., Nagafuji, S., Yamauchi, T., Okabe, H., Maki, J., Higo, H., Akahane, H., Aguilar, A., Jiménez-Estrada, M., & Reyes-Chilpa, R. (2002). Trypanocidal Constituents in Plants 1. Evaluation of Some Mexican Plants for Their Trypanocidal Activity and Active Constituents in Guaco, Roots of Aristolochia taliscana. Biological and Pharmaceutical Bulletin, 25(9), 1188–1191. https://doi.org/10.1248/BPB.25.1188	Plant used
Vigneron, M., Deparis, X., Deharo, E., & Bourdy, G. (2005). Antimalarial remedies in French Guiana: A knowledge attitudes and practices study. Journal of Ethnopharmacology, 98(3), 351–360. https://doi.org/10.1016/j.jep.2005.01.049	Study design
Betoni, J. E., Mantovani, R. P., Barbosa, L. N., di Stasi, L. C., Fern, & es Junior, A. (2006). Synergism between plant extract and antimicrobial drugs used on Staphylococcus aureus diseases. Mem Inst Oswaldo Cruz, 101(4), 387–390. https://doi.org/10.1590/s0074-02762006000400007	Plant mixture
Duarte, M. C. T., Leme, E. E., Delarmelina, C., Soares, A. A., Figueira, G. M., & Sartoratto, A. (2007). Activity of essential oils from Brazilian medicinal plants on Escherichia coli. Journal of Ethnopharmacology, 111(2), 197–201. https://doi.org/10.1016/j.jep.2006.11.034	Not an extract
Vigo, S. C., Maringoni, A. C., Camara, R. C., & Lima, G. P. P. (2009). Action of medicinal plants tinctures and essential oils to the bean common bacterial blight and on protein production of resistance induction. Summa Phytopathologica, 35(4), 293–304. https://doi.org/10.1590/s0100-54052009000400007	Not an extract
Alvarenga, F. C. R., Garcia, E. D. F., Bastos, E. M. A. F., Gr, i, T. S. M., & Duarte, M. G. R. (2009). Evaluation of the quality of commercial samples of leaves and tinctures of guaco. Revista Brasileira de Farmacognosia, 19(2), 442–448. https://doi.org/10.1590/S0102-695X2009000300018	Study design
Calderón, Á. I., Romero, L. I., Ortega-Barría, E., Solís, P. N., Zacchino, S., Gimenez, A., Pinzón, R., Cáceres, A., Tamayo, G., Guerra, C., Espinosa, A., Correa, M., & Gupta, M. P. (2010). Screening of Latin American plants for antiparasitic activities against malaria, Chagas disease, and leishmaniasis. Pharmaceutical Biology, 48(5), 545–553. https://doi.org/10.3109/13880200903193344	Plant used

de Paula, R. C., Sanchez, E. F., Costa, T. R., Martins, C. H. G., Pereira, P. S., Lourenço, M. v, Soares, A. M., & Fuly, A. L. (2010). Antiophidian properties of plant extracts against Lachesis muta venom. Journal of Venomous Animals and Toxins Including Tropical Diseases, 16(2), 311–323. https://doi.org/10.1590/s1678-91992010000200012	Part of the plant used
Bersan, S. M. F., Galvão, L. C. C., Goes, V. F. F., Sartoratto, A., Figueira, G. M., Rehder, V. L. G., Alencar, S. M., Duarte, R. M. T., Rosalen, P. L., & Duarte, M. C. T. (2014). Action of essential oils from Brazilian native and exotic medicinal species on oral biofilms. BMC Complementary and Alternative Medicine, 14(1). https://doi.org/10.1186/1472-6882-14-451	Not an extract

Table S3. Main characteristics of the included records for *M. glomerata*

**Author (s)**	**Country**	**Biome**	**Extraction process**	**Solvent**	**Method**	**Investigated biological activity**	**Results**
Zamprogno et al., 2015[1]	Brazil	Cerrado	Percolation	EtOH 96%	*In vitro*	Anthelmintics	Positive (*T. canis* and *A. caninum*.)
Fierro et al., 1999[2]	Brazil	Atlantic Forest	Maceration	EtOH and Dichloromethane	*In vivo*	Anti-allergic	Positive
Moreira et al., 2016[3]	NR	NR	Exhaustive extraction	Dichloromethane	*In vitro*	Antibiofilm	Positive
Lessa et al., 2012[4]	Brazil	Cerrado	Percolation	EtOH	*In vitro*	Antibiofilm	Positive
Maiorano et al., 2005[5]	Brazil	Atlantic Forest	Hot Extraction and Maceration	Water	*In vivo*	Anti-hemorrahagic	Positive
Mourão et al., 2014[6]	Brazil	Atlantic Forest	Maceration	EtOH 70%	*In vivo*	Anti-hemorrhagic	Positive
Borghi et al., 2023[7]	Brazil	Atlantic Forest	Ultrasound	EtOH 70%	*In vitro*	Anti-inflammatory	Negative
Moraes et al., 1994[8]	Brazil	Atlantic Forest	Maceration	EtOH 55%	*In vitro*	Anti-inflammatory	Positive
Paula et al., 2015[9]	Brazil	Amazonia	Maceration	EtOH 70%	*In vitro*	Anti-inflammatory	Positive

Author (s)	Country	Biome	Extraction process	Solvent	Method	Investigated biological activity	Results
Maiorano et al., 2005[5]	Brazil	Atlantic Forest	Hot Extraction and Maceration	Water	*In vivo*	Anti-inflammatory	Negative
Moura et al., 2002[10]	Brazil	Atlantic Forest	Infusion, decoction maceration	Water and EtOH	*in vivo*	Anti-inflammatory	Positive
Fierro et al., 1999[2]	Brazil	Atlantic Forest	Maceration and liquid-liquid fractionation	EtOH and a fraction of dichloromethane	*In vivo*	Anti-inflammatory	Positive only for immunologic inflammation
Floriano et al., 2009[11]	Brazil	Atlantic Forest	NR	Water	*In vivo*	Anti-inflammatory	Positive
Motta et al., 2017[12]	NR	NR	NR	Water	*In vivo*	Anti-inflammatory	Positive
Mourão et al., 2014[6]	Brazil	Atlantic Forest	Maceration	EtOH 70%	*In vivo*	Anti-inflammatory	Positve
Ruppelt et al., 1991[13]	Brazil	Atlantic Forest	Decoction	Water	*In vivo*	Anti-inflammatory	Positive
Della Pasqua et al., 2019[14]	Brazil	Atlantic Forest	Ultrasound	Water	*In vivo*	Anti-inflammatory	Positive
Freitas et al., 2008[15]	Brazil	Cerrado	Maceration	EtOH 70%	*In vivo*	Anti-inflammatory	Negative
Santos et al, 2006[16]	Brazil	Atlantic Forest	Percolation	EtOH-water 1:2 and water	*In vivo*	Anti-inflammatory	Positive
Bouzada e al., 2009[17]	Brazil	Atlantic Forest	Maceration	MeOH	*In vitro*	Antimicrobial	Positive for *P. aeruginosa, S. typhimurium, K. pneumoniae, B. cereus* and *E. coli*
Duarte et al, 2005[18]	Brazil	Atlantic Forest	Maceration	EtOH 70%	*In vitro*	Antimicrobial	Negative

Author (s)	Country	Biome	Extraction process	Solvent	Method	Investigated biological activity	Results
Holetz et al., 2002[19]	Brazil	Atlantic Forest	Maceration	EtOH 90%	*In vitro*	Antimicrobial	Negative
Moreira et al., 2016[3]	NR	NR	Exhaustive extraction	Dichloromethane	*In vitro*	Antimicrobial	Positive
Moreti et al., 2017[20]	Brazil	Atlantic Forest	Exhaustive extraction	Dichloromethane	*In vitro*	Antimicrobial	Positive for *P. gingivalis, P. acnes, P. nigrescens, P. melaninogenica, Aggregatibacter, actinomycetemcomitans*
Yatsuda et al., 2005[21]	Brazil	Atlantic Forest	Maceration	EtOH 70%	*In vitro*	Antimicrobial	Positive (*S. mutans, S. sobrinus, S. cricetus)*
Lessa et al., 2012[4]	Brazil	Cerrado	Percolation	EtOH	*In vitro*	Antimicrobial	Positive
Santana et al., 2019[22]	Brazil	Amazonia	Maceration	EtOH 70%	*In vivo*	Antimutagenic	Positive
Barbosa et al. 2012[23]	Brazil	NR	Maceration	EtOH70%	*In vivo*	Antimutagenic	Negative
Borghi et al., 2023[7]	Brazil	Atlantic Forest	Ultrasound	EtOH 70%	*In vitro*	Antioxidant	Positive
Chaves et al., 2020[24]	NR	NR	Infusion	Tea	*In vitro*	Antioxidant	Positive
Santana et al., 2014[25]	Brazil	Caatinga	Percolation	EtOH 70%	*In vitro*	Antioxidant	Positive
Nora et al.,2010[26]	Brazil	Atlantic Forest and Pampa	Infusion	water	*In vitro*	Antiproliferative	Positive
Holetz et al, 2002[27]	Brazil	Atlantic Forest	Maceration	EtOH 90%	*In vitro*	Antiprotozoal	Negative

Author (s)	Country	Biome	Extraction process	Solvent	Method	Investigated biological activity	Results
Luize et al., 2005[28]	Brazil	Atlantic Forest	Maceration	EtOH 90%	*In vitro*	Antiprotozoal	Positive
Floriano et al., 2009[11]	Brazil	Atlantic Forest	NR	Water	*In vivo*	Anti-sedation	Positive
Aboy et al., 2002[29]	Brazil	Atlantic Forest	Reflux, hot, cold, percolation	EtOH 96% and 50%	*Ex vivo*	Antispasmodic	Positive
Kaziyama et al., 2012[30]	NR	NR	Cold Extraction	Water	*In vitro*	Antiviral	Positive
Silva et al., 2016[31]	Brazil	Atlantic Forest	Maceration and percolation	EtOH 70%	*In vitro*	Antiviral	Positive
Santana et al., 2014[25]	Brazil	Caatinga	Percolation	EtOH 70%	*In vivo*	Anxiolytic	Positive
Moura et al., 2002[10]	Brazil	Atlantic Forest	Infusion, decoction maceration	Water and EtOH	*Ex vivo*	Bronchodilator	Positive
Garcia et al., 2020[32]	NR	NR	Turbolysis	EtOH and water	RCT	Bronchodilator	Negative
Borghi et al., 2023[7]	Brazil	Atlantic Forest	Ultrasound	EtOH 70%	*In vitro*	Cytotoxicity	Negative
Bouzada e al., 2009[17]	Brazil	Atlantic Forest	Maceration	MeOH	*In vitro*	Cytotoxicity	Positive
Luize et al., 2005[28]	Brazil	Atlantic Forest	Maceration	EtOH 90%	*In vitro*	Cytotoxicity	Negative
Kaziyama et al., 2012[30]	NR	NR	Cold Extraction	Water	*In vitro*	Cytotoxicity	Positive
Silva et al., 2016[31]	Brazil	Atlantic Forest	Maceration and percolation	EtOH 70%	*In vitro*	Cytotoxicity	Negative

Author (s)	Country	Biome	Extraction process	Solvent	Method	Investigated biological activity	Results
Moreira et al. 2016[3]	NR	NR	Exhaustive extraction	Dichloromethane	*In vivo*	Cytotoxicity	Negative
Costa et al., 2008[33]	Brazil	Atlantic Forest	Infusion and maceration	EtOH 80%	*In vitro*	Genotoxicity	Positive
Nora et al.,2010[26]	Brazil	Atlantic Forest and Pampa	Infusion	water	*In vitro*	Genotoxicity	Positve
Santos et al., 2019[34]	Brazil	Mangrove	Maceration	Water	*In vitro*	Genotoxicity	Positive
Moreira et al., 2016[3]	NR	NR	Exhaustive extraction	Dichloromethane	*In vivo*	Genotoxicity	Negative
Barbosa et al. 2012[23]	Brazil	NR	Maceration	EtOH70%	*In vivo*	Genotoxicity	Negative
Moura et al., 2002[10]	Brazil	Atlantic Forest	Infusion, decoction maceration	Water and EtOH	*In vitro*	Genotoxicity	Negative
Luize et al., 2005[28]	Brazil	Atlantic Forest	Maceration	EtOH 90%	*In vitro*	Hemolytic	Positive
Maiorano et al., 2005[5]	Brazil	Atlantic Forest	Hot Extraction and Maceration	Water	*In vitro*	Hemolytic	Negative
Chaves et al., 2020[24]	NR	NR	Infusion	Tea	*In vivo*	Hepatoprotective	Positive
Santana et al., 2014[25]	Brazil	Caatinga	Percolation	EtOH 70%	*In vivo*	Muscle relaxant	Negative
Souza et al., 2014[35]	Brazil	Atlantic Forest	Immersion	Water	*In vivo*	Molluscicidal	Positive (*Subulina octona)*
Costa et al., 2008[33]	Brazil	Atlantic Forest	Infusion and maceration	EtOH 80%	*In vitro*	Mutagenic	Negative

Author (s)	Country	Biome	Extraction process	Solvent	Method	Investigated biological activity	Results
Sá et al., 2006[36]	Brazil	Atlantic Forest	NR	EtOH 70%	*In vivo*	Mutagenic	Negative
Slomp et al., 2009[37]	Brazil	Atlantic Forest	Maceration	EtOH	*In vitro*	Nematocidal	Positive (Pratylenchus jaehni and Pratylenchus zeae)
Sá et al., 2010[38]	Brazil	Atlantic Forest	NR	EtOH 90%	*In vivo*	reproductive toxicity	Negative
Sá et al., 2006[36]	Brazil	Atlantic Forest	NR	EtOH 70%	*In vivo*	reproductive toxicity	Negative
Sá et al, 2003[39]	Brazil	Atlantic Forest	NR	EtOH 90%	*In vivo*	reproductive toxicity	Negative
Santana et al., 2014[25]	Brazil	Caatinga	Percolation	EtOH 70%	*In vivo*	Sedative	Negative
Santana et al., 2014[40]	Brazil	Caatinga	Maceration and percolation	EtOH 70%	*In vivo*	Toxicity	Negative
Bertol et al, 2024[41]	Brazil	Atlantic Forest	Ultrasound	EtOH 70%	RCT	Toxicity	Negative
Moura et al., 2002[10]	Brazil	Atlantic Forest	Infusion, decoction maceration	Water and EtOH	*Ex vivo*	Vasodilator	Positive

S4. Main characteristics of the included records for *M. laevigata*

Author (s)	Country	biome	Extraction process	Solvent	Method	Biological activity	Results
Zamprogno et al., 2015[1]	Brazil	Cerrado	Percolation	EtOH 96%	*In vitro*	anthelmintic	Positive
Massunari et al., 2020[42]	Brazil	Amazonia	Maceration	Hexane, ethyl acetate, and n-butanol: water (1:1, v/v)	*In vitro*	antibiofilm	Positive for E. faecalis, P. aeruginosa, and A. israelii
Lessa et al., 2012[4]	Brazil	Cerrado	Percolation	EtOH	*In vivo*	Antibiofilm	Positive
Leite et al.,2019[43]	Brazil	Cerrado	Maceration	EtOH	*In vitro*	Anticoagulant	Positive
Borghi et al., 2023[7]	Brazil	Atlantic Forest	Ultrasound	EtOH 70%	*In vitro*	Anti-inflammatory	Negative
Paula et al., 2015[9]	Brazil	Amazonia	Maceration	EtOH 70%	*In vitro*	Anti-inflammatory	Positive
Alves et al., 2009[44]	Brazil	Cerrado	Maceration	EtOH 70%	*In vitro*	Anti-inflammatory	Positive
Benatti et al., 2012[45]	Brazil	Cerrado	Maceration	EtOH 65%	*In vivo*	Anti-inflammatory	Positive
Pedroso et al., 2008[46]	Brazil	Atlantic Forest	Percolation	EtOH-water 1:2	*In vivo*	Anti-inflammatory	Positive
Santos et al, 2006[16]	Brazil	Atlantic Forest	Percolation	EtOH-water 1:2 and water	*In vivo*	Anti-inflammatory	Positive
Suyenaga et al., 2002[47]	Brazil	Atlantic Forest	Decoction	Water	*In vivo*	Anti-inflammatory	Positive
Della Pasqua et al., 2019[14]	Brazil	Atlantic Forest	Ultrasound	Water	*In vivo*	Anti-inflammatory	Positive
Freitas et al., 2008[15]	Brazil	Cerrado	Maceration	EtOH 70%	*In vivo*	Anti-inflammatory	Positive
Collaço et al., 2012[48]	NR	NR	NR	EtOH	*Ex vivo*	Anti-inflammatory	Positive
Baratto et al., 2008[49]	Brazil	Atlantic Forest	Maceration	EtOH 96%	*In vitro*	Antimicrobial	Negative
Massunari et al., 2020[50]	Brazil	Amazonia	Maceration	Hexane, ethyl acetate, and n-butanol: water (1:1, v/v)	*In vitro*	antimicrobial	Positive (ethyl acetate fraction)

Author (s)	Country	biome	Extraction process	Solvent	Method	Biological activity	Results
Duarte et al, 2005[18]	Brazil	Atlantic Forest	Maceration	EtOH 70%	*In vitro*	Antimicrobial	Negative
Yatsuda et al., 2005[21]	Brazil	Atlantic Forest	Maceration	EtOH 70%	*In vitro*	Antimicrobial	Positive
Lessa et al., 2012[4]	Brazil	Cerrado	Percolation	EtOH	*In vitro*	Antimicrobial	Positive
Fernandes et al., 2003[51]	Brazil	Pampa	Infusion	Water	*In vitro*	antimutagenic	Positive
Freitas et al., 2009[52]	Brazil	Amazonia	Maceration	EtOH 70%	*In vivo*	Antimutagenic	Negative
Mazzorana et al., 2013[50]	Brazil	Amazonia	Maceration	EtOH 70%	*In vivo*	Antimutagenic	Positive
Melo et al., 2009[53]	Brazil	Atlantic Forest	Percolation	EtOH 50%	*Ex vivo*	Anti-myotoxic	Positive
Collaço et al., 2012[48]	NR	NR	NR	EtOH	*Ex vivo*	Anti-neurotoxicity and Anti-myotoxic	Positive
Borghi et al., 2023[7]	Brazil	Atlantic Forest	Ultrasound	EtOH 70%	*In vitro*	Antioxidant	Positive
Ruffato et al., 2013[54]	Brazil	Atlantic Forest	Maceration	Hexane, ethyl acetate, chloroform, and EtOH-water (1:1, v/v)	*In vitro*	Antitumoral	Positive
Bighetti et al., 2005[55]	Brazil	Atlantic Forest	Maceration	EtOH 70%	*In vivo*	Antiulcerogenic	Positive
Pinto et al., 2017[56]	Brazil	Cerrado	Percolation	EtOH 80%	*In vivo*	Antiulcerogenic	Positive
Graça et al., 2007[57]	Brazil	Atlantic Forest	Maceration	EtOH 70%	*Ex vivo*	Bronchodilator	Positive
Ruffato et al., 2013[54]	Brazil	Atlantic Forest	Maceration	Hexane, ethyl acetate, chloroform, and EtOH-water (1:1, v/v)	*In vitro*	Cytotoxity	Negative
Freitas et al., 2009[52]	Brazil	Amazonia	Maceration	EtOH 70%	*In vivo*	Genotoxity	Negative

**Author (s)**	**Country**	**biome**	**Extraction process**	**Solvent**	**Method**	**Biological activity**	**Results**
Mazzorana et al., 2013[50]	Brazil	Amazonia	Maceration	EtOH 70%	*In vivo*	Genotoxity	Negative
Baratto et al., 2008[49]	Brazil	Atlantic Forest	Maceration	EtOH 96%	*In vitro*	Germination inhibitory	Positive
Fernandes et al., 2003[51]	Brazil	Pampa	Infusion	Water	*In vitro*	Mutagenic	Negative
Graça et al., 2007[58]	Brazil	Atlantic Forest	Percolation and maceration	EtOH 33ºGL	*In vivo*	Reproductive toxicity	Negative
Graça et al., 2007[57]	Brazil	Atlantic Forest	Maceration	EtOH 70%	*In vivo*	Toxicity	Negative
Bertol et al, 2024[41]	Brazil	Atlantic Forest	Ultrasound	EtOH 70%	RCT	Toxicity: adverse effects, blood markers and blood pressure	Negative

S5. Risk of bias (rob2) for randomized clinical trials

Study	Outcome	arising from the randomization process	effect of assignment to intervention	effect of adhering to intervention	missing outcome data	measurement of the outcome	selection of the reported result	Overall
Garcia et al., 2020[32]	Bronchodilator	low	low	low	low	low	low	Low
Bertol et al, 2024[41]	Adverse effects	low	low	low	low	Some concerns[1]	low	Some concerns

Bertol et al, 2024[41]	Blood pressure	low	low	low	low	low	low	Low
Bertol et al, 2024[41]	Blood paramaters	low	low	low	low	low	low	Low

1. The study was not blinded and the outcome is subjective

S6. Risk of bias (syrcle) for *in vivo studies*

Study	Selection			Performance		Detection		Attrition	Reporting	Other sources of bias	Overall
	**Sequence generation**	**Baseline characteristics**	**Allocation concealment**	**Random housing**	**blinding**	**Random outcome assessment**	**blinding**	**Incomplete outcome data**	**Selective outcome reporting**		
Alves et al., 2009[44]	Unclear[1]	Low	Unclear[3]	Unclear[4]	Unclear[5]	Unclear[6]	Unclear[7]	Unclear[9]	low	low	Unclear
Barbosa et al., 2012[23]	Unclear[1]	Low	Unclear[3]	Low	Low	Unclear[6]	Unclear[7]	High[8]	Low	Low	High
Benatti et al., 2012[45]	High[11]	Low	Unclear[3]	Unclear[4]	Unclear[5]	Unclear[6]	Unclear[7]	Unclear[9]	Unclear[10]	low	High
Bighetti et al., 2005[55]	Unclear[1]	Low	Unclear[3]	Low	Unclear[5]	Unclear[6]	Unclear[7]	Unclear[9]	Low	Low	Unclear
Chaves et al., 2020[24]	Unclear[1]	Low	Unclear[3]	Low	Low	Unclear[6]	Unclear[7]	Low	Low	Low	Unclear
Della Pasqua et al., 2019[14]	Unclear[1]	low	Unclear[3]	Low	low	Unclear[6]	Unclear[7]	low	low	low	Unclear
Fierro et al., 1999[2]	Unclear[1]	Low	Unclear[3]	Unclear[4]	Low	Unclear[6]	Unclear[7]	High[8]	low	Low	Unclear
Floriano et al., 2009[11]	Unclear[1]	Low	Unclear[3]	Low	Low	Unclear[6]	Unclear[7]	Low	Low	Low	Unclear

Study	Selection			Performance		Detection		Attrition	Reporting	Other sources of bias	Overall
	Sequence generation	Baseline characteristics	Allocation concealment	Random housing	blinding	Random outcome assessment	blinding	Incomplete outcome data	Selective outcome reporting		
Freitas et al., 2008[15]	Unclear[1]	Unclear[2]	Unclear[3]	Low	low	Unclear[6]	Unclear[7]	low	low	low	Unclear
Freitas et al., 2009[52]	Unclear[1]	Unclear[2]	Unclear[3]	Low	low	Unclear[6]	Unclear[7]	Unclear[9]	Unclear[10]	low	Unclear
Graça et al., 2007[57]	Unclear[1]	Low	Unclear[3]	Low	Low	Unclear[6]	Unclear[7]	low	low	low	Unclear
Graça et al., 2007[58]	Unclear[1]	Low	Unclear[3]	Low	Low	Unclear[6]	Unclear[7]	low	low	low	Unclear
Maiorano et al., 2005[5]	Unclear[1]	Low	Unclear[3]	Low	low	Unclear[6]	Unclear[7]	High[8]	Low	Low	High
Mazzorana et al., 2013[50]	Unclear[1]	low	Unclear[3]	Low	low	Unclear[6]	Unclear[7]	Low	low	Low	Unclear
Motta et al., 2017[12]	Unclear[1]	Low	Unclear[3]	Low	Low	Unclear[6]	Unclear[7]	Low	Low	Low	Unclear
Moura et al., 2002[10]	High[11]	Low	Unclear[3]	Unclear[4]	Unclear[5]	Unclear[6]	Unclear[7]	Low	Unclear[10]	Low	High
Mourão et al., 2014[6]	Unclear[1]	Low	Unclear[3]	Low	Unclear[5]	Unclear[6]	Unclear[7]	Low	Low	Low	Unclear
Pedroso et al., 2008[46]	Unclear[1]	low	Unclear[3]	Low	low	Unclear[6]	Unclear[7]	Unclear[9]	low	low	Unclear
Pinto et al., 2017[56]	High[11]	Low	Unclear[3]	Low	Unclear[5]	Unclear[6]	Unclear[7]	low	low	low	High
Ruppelt et al., 1991[13]	Unclear[1]	low	Unclear[3]	Unclear[4]	Low	Unclear[6]	Unclear[7]	Low	Low	Low	Unclear
Sá et al, 2003[39]	Unclear[1]	low	Unclear[3]	Low	Low	Unclear[6]	Unclear[7]	Low	low	Low	Unclear

Study	Selection			Performance		Detection		Attrition	Reporting	Other sources of bias	Overall
	**Sequence generation**	**Baseline characteristics**	**Allocation concealment**	**Random housing**	**blinding**	**Random outcome assessment**	**blinding**	**Incomplete outcome data**	**Selective outcome reporting**		
Sá et al., 2006[36]	Unclear[1]	Low	Unclear[3]	Unclear[4]	Unclear[5]	Unclear[6]	Unclear[7]	Low	Low	Low	Unclear
Sá et al., 2010[38]	Unclear[1]	Low	Unclear[3]	Low	Low	Unclear[6]	Unclear[7]	High[8]	Unclear[10]	Low	High
Santana et al., 2014[25]	Unclear[1]	low	Unclear[3]	Low	low	Unclear[6]	Unclear[7]	low	low	low	Unclear
Santana et al., 2014[40]	Unclear[1]	low	Unclear[3]	Low	low	Unclear[6]	Unclear[7]	low	low	low	Unclear
Santana et al., 2019[22]	Unclear[1]	low	Unclear[3]	Low	Low	Unclear[6]	Unclear[7]	Low	low	low	Unclear
Santos et al, 2006[16]	High[11]	low	Unclear[3]	Low	Unclear[5]	Unclear[6]	Unclear[7]	Unclear[9]	Unclear[10]	low	High
Souza et al., 2014[35]	Unclear[1]	low	Unclear[3]	Low	low	Unclear[6]	Unclear[7]	low	low	low	Unclear
Suyenaga et al., 2002[47]	Unclear[1]	low	Unclear[3]	Low	Unclear[5]	Unclear[6]	Unclear[7]	Unclear[9]	low	low	Unclear

1. Does not report how the allocation sequence was generated
2. Does not report the animals' baseline data
3. Does not report how the different groups were allocated
4. No information on how the rats were housed
5. Does not inform about blinding of caregivers and/or investigators, and are a subjective outcome
6. No information on how the animals were chosen for assessment
7. Does not inform about blinding of caregivers and/or assessor
8. The final n is higher than the initial n or 10% lower without justification
9. Did not inform the number of animals evaluated

10. Not all of the study's pre-specified primary outcomes have been reported

S7. Characteristics of tests with negative results for *M. glomerata*

Investigated biological activity	Method	Model/Assay/inclusion criteria	Solvent	Author (s)
Hemolytic	In vitro	agarose–egg yolk–erythrocyte gels as substrate.	Water	Maiorano et al., 2005[5]
Anti-inflammatory	In vitro	Dystrophic Primary Skeletal Muscle Cell Culture	EtOH 70%	Borghi et al., 2023[7]
	In vivo	Mice with edema induceed by Bothrops jararacussu and Crotalus durissus terriflcus venoms	Water	Maiorano et al., 2005[5]
		BALB/c mice with allergic pneumonitis sensitized with ovalbumin and aluminum oxide.	EtOH-water 1:2 and water	Santos et al, 2006[46]
Antimicrobial	In vitro	Culture of Candida Albicans	EtOH 70%	Duarte et al, 2005[18]
		Culture of Escherichia coli, Pseudomonas aeruginosa, Bacillus subtilis, Staphylococcus aureus, Candida albicans, C. krusei, C. parapsilosis, and C. tropicalis.	EtOH 90%	Holetz et al., 2002[19]
Antimutagenic	In vivo	Mice on on DXR-induced mutagenicity	EtOH70%	Barbosa et al. 2012[23]
Antiprotozoal	In vitro	Herpetomonas samuelpessoai	EtOH 90%	Holetz et al, 2002[27]
		Trypanosoma cruzi	EtOH 90%	Luize et al., 2005[28]
Bronchodilator	RCT	Volunteers with mild or moderate persistent asthma	EtOH and water	Garcia et al., 2020[32]
Cytotoxicity	In vitro	neutral red assay	EtOH 70%	Borghi et al., 2023[7]
		Red blood cell (RBC) lysis assay	EtOH 90%	Luize et al., 2005[28]
		Vero cells	EtOH 70%	Silva et al., 2016[31]
	In vivo	Swiss mice treat with KAMg	Dichloromethane	Moreira et al. 2016[3]
Genotoxicity	In vivo	Swiss mice treat with KAMg	Dichloromethane	Moreira et al., 2016[3]
		Mice on DXR-induced mutagenicity	EtOH70%	Barbosa et al. 2012[23]
	In vitro	plasmid DNA using an alkaline lyses procedure	Water and EtOH	Moura et al., 2002[10]
Muscle relaxant	In vivo	Albino Swiss mice	EtOH 70%	Santana et al., 2014[25]
Mutagenic	In vitro	The alkylating and clastogenic induce DNA damage in rat hepatoma cells	EtOH 80%	Costa et al., 2008[33]
reproductive toxicity	In vivo	Male Wistar rats	EtOH 70%	Sá et al., 2006[36]
	In vivo	Male Wistar rats	EtOH 90%	Sá et al., 2010[38]
	In vivo	Male Wistar rats	EtOH 70%	Sá et al., 2006[36]
	In vivo	Male Wistar rats	EtOH 90%	Sá et al, 2003[39]
Sedative	In vivo	Albino Swiss mice	EtOH 70%	Santana et al., 2014[25]
Toxicity	In vivo	Swiss mice	EtOH 70%	Santana et al., 2014[40]
Toxicity	RCT	Health volunteers	EtOH 70%	Bertol et al, 2024[41]

S8. Characteristics of tests with negative results for *M. laevigata*

Investigated biological activity	Method	Model/Assay/inclusion criteria	Solvent	Author (s)
Anti-inflammatory	*In vitro*	Dystrophic Primary Skeletal Muscle Cell Culture	EtOH 70%	Borghi et al., 2023[7]
Antimicrobial	*In vitro*	Culture of Staphylococcus aureus, Escherichia coli, Pseudomonas aeruginosa, Enterococcus faecalis and Enterococcus faecium.	EtOH 96%	Baratto et al., 2008[49]
	In vitro	Culture of Candida Albicans	EtOH 70%	Duarte et al, 2005[17]
Antimutagenic	*In vivo*	Wistar treat with coal dust directly in the lung	EtOH 70%	Freitas et al., 2009[52]
Cytotoxity	*In vitro*	non tumor (MRC-5) cell lines	Hexane, ethyl acetate, chloroform, and EtOH-water (1:1, v/v)	Ruffato et al., 2013[54]
Genotoxity	*In vivo*	Wistar treat with coal dust directly in the lung	EtOH 70%	Freitas et al., 2009[52]
	In vivo	CF1 mice	EtOH 70%	Mazzorana et al., 2013[50]
Mutagenic	*In vitro*	Salmonella/microsome assay	Water	Fernandes et al., 2003[51]
Reproductive toxicity	*In vivo*	Male wistar rats	EtOH 33ºGL	Graça et al., 2007[58]
Toxicity	*In vivo*	Wistar rats and Swiss mice	EtOH 70%	Graça et al., 2007[57]
Toxicity: adverse effects, blood markers and blood pressure	RCT	Health volunteers	EtOH 70%	Bertol et al, 2024[40]

REFERENCES

1. Zamprogno TT, Garcia Lopes ADC, Lacerda T, Hiura E, Da Fonseca LA, Senna T, Soares FEF, Endringer DC, Araujo JV, et al. Activity of Euterpe edulis martius, Mikania glomerata spreng, and Mikania laevigata schultz bip. Extracts on gastrointestinal nematodes toxocara canis and ancylostoma caninum. *Archives of Clinical Infectious Diseases*. **2015**, 10.
2. Fierro IM, Borges Da Silva AC, Da Silva Lopes C, Soares De Moura R, Barja-Fidalgo C. Studies on the anti-allergic activity of Mikania glomerata. *Journal of Ethnopharmacology*. **1999**, 66, 19-24.
3. Moreira MR, Souza AB, Soares S, Bianchi TC, De Souza Eugênio D, Lemes DC, Martins CHG, Da Silva Moraes T, Tavares DC, et al. ent-Kaurenoic acid-rich extract from Mikania glomerata: In vitro activity against bacteria responsible for dental caries. *Fitoterapia*. **2016**, 112, 211-216.

4. Lessa FCR, Grillo CHB, Pinto FE, Lorençon BB, Martins JDL, Bertolucci SKV, Pinto JEBP, Endringer DC. Efficacy of guaco mouthwashes (Mikania glomerata and Mikania laevigata) on the disinfection of toothbrushes. *Revista Brasileira de Farmacognosia*. **2012**, 22, 1330-1337.
5. Maiorano VA, Marcussi S, Daher MA, Oliveira CZ, Couto LB, Gomes OA, França SC, Soares AM, Pereira PS. Antiophidian properties of the aqueous extract of Mikania glomerata. *J Ethnopharmacol*. **2005**, 102, 364-370.
6. Mourão VB, Giraldi GM, Neves LMG, de Gaspari de Gaspi FO, Rodrigues RAF, Alves AA, Esquisatto MAM, Mazzi MV, Mendonça FAS, et al. Anti-hemorrhagic effect of hydro-alcoholic extract of the leaves of Mikania glomerata in lesions induced by Bothrops jararaca venom in rats. *Acta cirurgica brasileira*. **2014**, 29, 30-37.
7. Borghi AA, Minatel E, Mizobuti DS, de Lourenço CC, Fernandes de Araújo F, Maria Pastore G, Hewitson P, Ignatova S, Chf Sawaya A. Antioxidant and Anti-inflammatory Activity of Mikania glomerata and Mikania laevigata Extracts. *Pharmacognosy Research*. **2023**, 15, 128-137.
8. Moraes VL, Santos LF, Castro SB, Loureiro LH, Lima OA, Souza ML, Yien LM, Rossi-Bergmann B, Costa SS. Inhibition of lymphocyte activation by extracts and fractions of Kalanchoe, Alternanthera, Paullinia and Mikania species. *Phytomedicine*. **1994**, 1, 199-204.
9. Chagas-Paula D, Oliveira T, Faleiro D, Oliveira R, Da Costa F. Outstanding Anti-inflammatory Potential of Selected Asteraceae Species through the Potent Dual Inhibition of Cyclooxygenase-1 and 5-Lipoxygenase. *Planta Medica*. **2015**, 81, 1296-1307.
10. de Moura R, Costa SS, Jansen JM, Silva CA, Lopes CS, Bernardo-Filho M, da Silva V, Criddle DN, Portela BN, et al. Bronchodilator activity of Mikania glomerata Sprengel on human bronchi and guinea-pig trachea. *J Pharm Pharmacol*. **2002**, 54, 249-256.
11. Floriano RS, Nogueira RM, Sakate M, Laposy CB, da Motta YP, Sangiorgio F, David HC, Nabas JM. Effect of Mikania glomerata (Asteraceae) leaf extract combined with anti-venom serum on experimental Crotalus durissus (Squamata: Viperidae) envenomation in rats. *Rev Biol Trop*. **2009**, 57, 929-937.
12. Motta YP, Sakate M, Nogueira RMB, Peraçoli MTS, Sangiorgio F, Floriano RS, Takahagi EN. Quantification of cytokines in serum and paw homogenate of experimental intoxication for venom of the Bothropoides jararaca in Wistar rats treated with antivenom and Mikania glomerata. *Arquivo Brasileiro de Medicina Veterinaria e Zootecnia*. **2014**, 66, 1413-1418.
13. Ruppelt BM, Pereira EFR, Goncalves LC, Pereira NA. Pharmacological screening of plants recommended by folk medicine as anti snake venom. I-Analgesic and anti-inflammatory activities. *Revista Brasileira de Farmacia*. **1990**, 71, 54-56.
14. Della Pasqua CDP, Antunes E, Sawaya A, Campos R, ucci ECT. Beneficial effects of aqueous extracts of Mikania glomerata Sprengel and Mikania laevigata Schultz Bip ex Baker on the inflammatory response in rats with acute pancreatitis. *Faseb Journal*. **2019**, 33.
15. Freitas TP, Silveira PC, Rocha LG, Rezin GT, Rocha J, Citadini-Zanette V, Romão PT, Dal-Pizzol F, Pinho RA, et al. Effects of Mikania glomerata Spreng. and Mikania laevigata Schultz Bip. ex Baker (Asteraceae) extracts on pulmonary inflammation and oxidative stress caused by acute coal dust exposure. *J Med Food*. **2008**, 11, 761-766.
16. dos Santos SC, Krueger CL, Steil AA, Kreuger MR, Biavatti MW, Wisniewski Junior A. LC characterisation of guaco medicinal extracts, Mikania laevigata and M. glomerata, and their effects on allergic pneumonitis. *Planta Med*. **2006**, 72, 679-684.
17. Bouzada MLM, Fabri RL, Nogueira M, Konno TUP, Duarte GG, Scio E. Antibacterial, cytotoxic and phytochemical screening of some traditional medicinal plants in Brazil. *Pharmaceutical Biology*. **2009**, 47, 44-52.

18. Duarte MC, Figueira GM, Sartoratto A, Rehder VL, Delarmelina C. Anti-Candida activity of Brazilian medicinal plants. *J Ethnopharmacol*. **2005**, 97, 305-311.
19. Holetz FB, Pessini GL, Sanches NR, Cortez DA, Nakamura CV, Filho BP. Screening of some plants used in the Brazilian folk medicine for the treatment of infectious diseases. *Mem Inst Oswaldo Cruz*. **2002**, 97, 1027-1031.
20. Moreti DLC, Leandro LF, da Silva Moraes T, Moreira MR, Sola Veneziani RC, Ambrosio SR, Figueiredo Almeida Gomes BP, Martins CHG. Mikania glomerata Sprengel extract and its major compound ent-kaurenoic acid display activity against bacteria present in endodontic infections. *Anaerobe*. **2017**, 47, 201-208.
21. Yatsuda R, Rosalen PL, Cury JA, Murata RM, Rehder VL, Melo LV, Koo H. Effects of Mikania genus plants on growth and cell adherence of mutans streptococci. *J Ethnopharmacol*. **2005**, 97, 183-189.
22. Santana EA, Rodrigues RF, de Almeida FB, Oliveira AEMdFM, Cruz RAS, França HS, Batitucci MdCP, Dutra JCV, da Silva Ferreira P, et al. Simultaneous extraction and obtention of a novel nano-dispersion from Mikania glomerata Spreng: Monitoring coumarin content and increasing the biological and industrial potential of a classical cultivated herb. *Industrial Crops and Products*. **2019**, 135, 49-56.
23. Barbosa LC, de Morais M, de Paula CA, da Silva Ferreira MC, Jordao AA, e Silva ML, Kenupp Bastos J, da Silva Filho AA, de Oliveira Cecchi A. Mikania glomerata Sprengel (Asteraceae) influences the mutagenicity induced by doxorubicin without altering liver lipid peroxidation or antioxidant levels. *J Toxicol Environ Health A*. **2012**, 75, 1102-1109.
24. Chaves PFP, Adami ER, Corso CR, Milani L, de Oliveira NMT, da Silva LCM, Acco A, Iacomini M, Cordeiro LMC. Carbohydrates from Mikania glomerata Spreng tea: Chemical characterization and hepatoprotective effects. *Bioactive Carbohydrates and Dietary Fibre*. **2020**, 24.
25. Santana LCLR, Brito MRM, Oliveira GLS, Citó AMGL, Alves CQ, David JP, David JM, De Freitas RM. Mikania glomerata: Phytochemical, Pharmacological, and Neurochemical Study. *Evidence-based Complementary and Alternative Medicine : eCAM*. **2014**, 2014.
26. Dalla Nora G, Pastori T, Laughinghouse HDt, Do Canto-Dorow TS, Tedesco SB. Antiproliferative and genotoxic effects of Mikania glomerata (Asteraceae). *Biocell*. **2010**, 34, 95-101.
27. Barbieri Holetz F, Ueda-Nakamura T, Dias Filho BP, Garcia Cortez DA, Palazzo Mello JC, Vataru Nakamura C. Effect of plant extracts used in folk medicine on cell growth and differentiation of Herpetomonas samuelpessoai (kinetoplastida, trypanosomatidae) cultivated in defined medium. *Acta Scientiarum - Biological and Health Sciences*. **2002**, 24, 657-662.
28. Luize PS, Tiuman TS, Morello LG, Maza PK, Ueda-Nakamura T, Dias Filho BP, Cortez DAG, De Mello JCP, Nakamura CV. Effects of medicinal plant extracts on growth of Leishmania (L.) amazonensis and Trypanosoma cruzi. *Revista Brasileira de Ciencias Farmaceuticas/Brazilian Journal of Pharmaceutical Sciences*. **2005**, 41, 85-94.
29. Aboy AL, Ortega GG, Petrovick PR, Langeloh A, Bassani VL. Antispasmodic activity of leaf extracts of Mikania glomerata sprengel (guaco). *Acta Farmaceutica Bonaerense*. **2002**, 21, 185-191.
30. Kaziyama VM, Fern, es MJB, Simoni IC. Antiviral activity of commercially available medicinal plants on suid and bovine herpesviruses. *Revista Brasileira de Plantas Medicinais*. **2012**, 14, 522-528.
31. Silva ME, Vilela FMP, Mir, a MA, Húngaro HM, Ferreira FM, Yamamoto CH, Cavalcanti JF, Romanos MTV, et al. Mikania glomerata extract inhibits herpes simplex virus type-1 (HSV-1) and type-2 (HSV-2). *Revista Brasileira de Plantas Medicinais*. **2016**, 18, 732-737.

32. Garcia CG, Leonart LP, Lenzi L, Bertol G, Gasparetto JC, Barros JA, Pontarolo R. Evaluation of the Bronchodilator Effect of Guaco Syrup: a Randomized, Double-blind, Crossover Clinical Trial. *Revista Brasileira de Farmacognosia*. **2020**, 30, 111-117.
33. Costa Rde J, Diniz A, Mantovani MS, Jordão BQ. In vitro study of mutagenic potential of Bidens pilosa Linné and Mikania glomerata Sprengel using the comet and micronucleus assays. *J Ethnopharmacol*. **2008**, 118, 86-93.
34. dos Santos RR, Turra B, Simon K, Damiani AP, Strapazzon G, Leandro RT, Vilela TC, Peterson M, de Andrade VM, et al. Evaluation of genotoxicity and coumarin production in conventional and organic cultivation systems of Mikania glomerata Spreng. *Journal of environmental science and health Part B, Pesticides, food contaminants, and agricultural wastes*. **2019**, 54, 866-874.
35. de Souza BA, da Silva LC, Chicarino ED, Bessa ECA. Phytochemical and molluscicidal activity of Mikania glomerata Sprengel (Asteraceae) in different lifestages of Subulina octona (Mollusca, Subulinidade). *Brazilian Archives of Biology and Technology*. **2014**, 57, 261-268.
36. Sá RDCDS, Leite MN, Peters VM, Guerra MDO, De Almeida RN. Absence of mutagenic effect of Mikania glomerata hydroalcoholic extract on adult wistar rats in vivo. *Brazilian Archives of Biology and Technology*. **2006**, 49, 599-604.
37. Slomp L, Pereira PS, De Castro França S, Zingaretti S, Beleboni RO. In vitro nematocidal effects of medicinal plants from são paulo state, brazil. *Pharmaceutical Biology*. **2009**, 47, 230-235.
38. da Silveira e Śa RC, Leite MN, de Almeida RN. Toxicological screening of Mikania glomerata Spreng., Asteraceae, extract in male Wistar rats reproductive system, sperm production and testosterone level after chronic treatment. *Revista Brasileira de Farmacognosia*. **2010**, 20, 718-723.
39. da Silveira e Sá Rde C, Leite MN, Reporedo Mde M, de Almeida RN. Evaluation of long-term exposure to Mikania glomerata (Sprengel) extract on male Wistar rats' reproductive organs, sperm production and testosterone level. *Contraception*. **2003**, 67, 327-331.
40. Santana LCLR, Brito MRM, Sousa GF, Freitas RM. Physicochemical properties and acute toxicity evaluation of ethanol extract of the leaves from Mikania glomerata Sprengel. *Revista Brasileira de Plantas Medicinais*. **2014**, 15, 742-750.
41. Bertol G, Cobre AdF, Campos ML, Pontarolo R. Safety evaluation of Mikania glomerata and Mikania laevigata in healthy volunteers: A randomized, open label and multiple dose phase I clinical trial. *Journal of ethnopharmacology*. **2024**, 318.
42. Massunari L, Souza ACA, Domingues PFK, Scardelato JA, Sacramento LVS, Dezan-Junior E, Duque C. Inhibitory activity of varronia curassavica and mikania laevigata fractions against pathogens associated with persistent dental infections. *Revista de Ciencias Farmaceuticas Basica e Aplicada*. **2020**, 41, 1-10.
43. Leite PM, Miranda APN, Amorim JM, Duarte RCF, Bertolucci SKV, Carvalho MDG, Castilho RO. In Vitro Anticoagulant Activity of Mikania laevigata: Deepening the Study of the Possible Interaction Between Guaco and Anticoagulants. *Journal of cardiovascular pharmacology*. **2019**, 74, 574-583.
44. Alves CF, Alves VB, de Assis IP, Clemente-Napimoga JT, Uber-Bucek E, Dal-Secco D, Cunha FQ, Rehder VL, Napimoga MH. Anti-inflammatory activity and possible mechanism of extract from Mikania laevigata in carrageenan-induced peritonitis. *J Pharm Pharmacol*. **2009**, 61, 1097-1104.
45. Benatti BB, Campos-Júnior JC, Silva-Filho VJ, Alves PM, Rodrigues IR, Uber-Bucek E, Vieira SM, Napimoga MH. Effects of a Mikania laevigata extract on bone resorption and RANKL expression during experimental periodontitis in rats. *J Appl Oral Sci*. **2012**, 20, 340-346.

46. Pedroso APD, Santos SC, Steil AA, Deschamps F, Barison A, Campos F, Biavatti MW. Isolation of syringaldehyde from Mikania laevigata medicinal extract and its influence on the fatty acid profile of mice. *Revista Brasileira de Farmacognosia*. **2008**, 18, 63-69.
47. Suyenaga ES, Reche E, Farias FM, Schapoval EE, Chaves CG, Henriques AT. Antiinflammatory investigation of some species of Mikania. *Phytother Res*. **2002**, 16, 519-523.
48. Collaço Rde C, Cogo JC, Rodrigues-Simioni L, Rocha T, Oshima-Franco Y, azzo-Moura P. Protection by Mikania laevigata (guaco) extract against the toxicity of Philodryas olfersii snake venom. *Toxicon*. **2012**, 60, 614-622.
49. Baratto L, Lang KL, Vanz DC, Reginatto FH, Oliveira JB, Falkenberg M. Investigation of the allelopathic and antimicrobial activities of Mikania laevigata (Asteraceae) obtained in hydroponic and traditional cultivars. *Revista Brasileira de Farmacognosia*. **2008**, 18, 577-582.
50. Medeiros Mazzorana D, Nicolau V, Moreira J, de Aguiar Amaral P, de Andrade VM. Influence of Mikania laevigata Extract over the Genotoxicity Induced by Alkylating Agents. *ISRN Toxicol*. **2013**, 2013, 521432-521432.
51. Fern, es JB, Vargas VM. Mutagenic and antimutagenic potential of the medicinal plants M. laevigata and C. xanthocarpa. *Phytother Res*. **2003**, 17, 269-273.
52. Freitas TP, Heuser VD, Tavares P, Leffa DD, da Silva GA, Citadini-Zanette V, Romão PR, Pinho RA, Streck EL, et al. Genotoxic evaluation of Mikania laevigata extract on DNA damage caused by acute coal dust exposure. *J Med Food*. **2009**, 12, 654-660.
53. Melo RS, Farrapo NM, Junior DSR, Silva MG, Cogo JC, Belo CAD, Rodrigues-Simioni L, Groppo FC, Oshima-Franco Y. Antiophidian mechanisms of medicinal plants. *Flavonoids: Biosynthesis, Biological Effects and Dietary Sources*. **2009**, 249-262.
54. Rufatto LC, Finimundy TC, Roesch-Ely M, Moura S. Mikania laevigata: chemical characterization and selective cytotoxic activity of extracts on tumor cell lines. *Phytomedicine*. **2013**, 20, 883-889.
55. Bighetti AE, Antônio MA, Kohn LK, Rehder VL, Foglio MA, Possenti A, Vilela L, Carvalho JE. Antiulcerogenic activity of a crude hydroalcoholic extract and coumarin isolated from Mikania laevigata Schultz Bip. *Phytomedicine*. **2005**, 12, 72-77.
56. Pinto M, Oliveira E, Martins J, De Paula J, Costa E, Da Conceição E, Bara M. Obtaining a Dry Extract from the Mikania laevigata Leaves with Potential for Antiulcer Activity. *Pharmacognosy Magazine*. **2017**, 13, 76-76.
57. Graça C, Baggio CH, Freitas CS, Rattmann YD, de Souza LM, Cipriani TR, Sassaki GL, Rieck L, Pontarolo R, et al. In vivo assessment of safety and mechanisms underlying in vitro relaxation induced by Mikania laevigata Schultz Bip. ex Baker in the rat trachea. *J Ethnopharmacol*. **2007**, 112, 430-439.
58. Graça C, Freitas CS, Baggio CH, Dalsenter PR, Marques MC. Mikania laevigata syrup does not induce side effects on reproductive system of male Wistar rats. *J Ethnopharmacol*. **2007**, 111, 29-32.
